# A non-randomized controlled trial to assess the protective effect of SMC in the context of high parasite resistance in Uganda

**DOI:** 10.1186/s12936-023-04488-4

**Published:** 2023-02-22

**Authors:** Anthony Nuwa, Kevin Baker, Craig Bonnington, Musa Odongo, Tonny Kyagulanyi, John Baptist Bwanika, Sol Richardson, Jane Nabakooza, Jane Achan, Richard Kajubi, David Salandini Odong, Maureen Nakirunda, Godfrey Magumba, Geofrey Beinomugisha, Madeleine Marasciulo-Rice, Hilda Abio, Christian Rassi, Damian Rutazaana, Denis Rubahika, James Tibenderana, Jimmy Opigo

**Affiliations:** 1 Malaria Consortium Uganda, Kampala, Uganda; 2 Malaria Consortium, London, UK; 3 Department of Global Public Health, Karolinska Institute, Stockholm, Sweden; 4 Vanke School of Public Health, Tsinghua University, Beijing, China; 5 National Malaria Control Division, Ministry of Health, Kampala, Uganda; 6 Malaria Consortium US, Raleigh, USA

**Keywords:** Season malaria chemoprevention, Protective efficacy, Malaria case, Malaria episode, Resistance markers

## Abstract

**Background:**

Until recently, due to widespread prevalence of molecular markers associated with sulfadoxine-pyrimethamine (SP) and amodiaquine (AQ) resistance in east and southern Africa, seasonal malaria chemoprevention (SMC) has not been used at scale in this region. This study assessed the protective effectiveness of monthly administration of SP + AQ (SPAQ) to children aged 3–59 months in Karamoja sub-region, Uganda, where parasite resistance is assumed to be high and malaria transmission is seasonal.

**Methods:**

A two-arm quasi-experimental, open-label prospective non-randomized control trial (nRCT) was conducted in three districts. In two intervention districts, 85,000 children aged 3–59 months were targeted to receive monthly courses of SMC using SPAQ during the peak transmission season (May to September) 2021. A third district served as a control, where SMC was not implemented. Communities with comparable malaria attack rates were selected from the three districts, and households with at least one SMC-eligible child were purposively selected. A total cohort of 600 children (200 children per district) were selected and followed using passive surveillance for breakthrough confirmed malaria episodes during the five-month peak transmission season. Malaria incidence rate per person-months and number of malaria episodes among children in the two arms were compared. Kaplan–Meier failure estimates were used to compare the probability of a positive malaria test. Other factors that may influence malaria transmission and infection among children in the two arms were also assessed using multivariable cox proportional hazards regression model.

**Results:**

The malaria incidence rate was 3.0 and 38.8 per 100 person-months in the intervention and control groups, respectively. In the intervention areas 90.0% (361/400) of children did not experience any malaria episodes during the study period, compared to 15% (29/200) in the control area. The incidence rate ratio was 0.078 (95% CI 0.063–0.096), which corresponds to a protective effectiveness of 92% (95% CI 90.0–94.0) among children in the intervention area.

**Conclusion:**

SMC using SPAQ provided high protective effect against malaria during the peak transmission season in children aged 3–59 months in the Karamoja sub-region of Uganda.

## Background

Malaria remains one of the most challenging infectious diseases globally, causing 241 million cases in 85 malaria endemic countries and 627,000 deaths in 2020 [1]. Uganda has one of the highest malaria burdens worldwide, ranking third (5.4%) and fifth (3.5%), for malaria morbidity and mortality, respectively [2]. For over two decades, the Karamoja sub-region in northeastern Uganda has consistently reported the highest malaria prevalence among the country’s regions. At the end of 2019, malaria prevalence was 34% compared to 9% nationally. Children under five years of age are disproportionately affected [3–5]. The region is primarily inhabited by nomadic pastoralists who live in small makeshift houses, which makes the use of conventional malaria control interventions such as long-lasting insecticidal nets and indoor residual spraying challenging [6]. In the 2021–2025 Uganda Malaria Reduction and Elimination Strategic Plan (UMRESP), the Ministry of Health (MOH) proposed piloting innovative malaria interventions, such as seasonal malaria chemoprevention (SMC), in this setting [6].

Several studies conducted in west and central Africa have shown that SMC, which involves the community-based, monthly administration of sulfadoxine-pyrimethamine (SP) and amodiaquine (AQ) to children 3–59 months during the peak malaria transmission season, is a highly effective intervention. It can prevent up to 75% of mild and severe malaria cases, as well as averting deaths in eligible children [7–10]. In 2012, the World Health Organization (WHO) recommend the scale-up of SMC in areas where malaria transmission is highly seasonal and the therapeutic efficacy of SP and AQ is above 90% [11]. Consequently, the Sahel region was prioritized for SMC, as prevalence of resistance makers for SP is high in much of east and southern Africa [12–15]. However, studies have shown that use of SP for intermittent preventive treatment in pregnancy (IPTp), another chemoprevention strategy, remained effective despite high parasite resistance [16, 17]. This has led to increased interest in exploring whether SMC using SPAQ could work in areas of east and southern Africa where malaria transmission is seasonal [18]. Data from Uganda’s National Malaria Control Division (NMCD) show that malaria transmission in the Karamoja sub-region is highest between May and September, with more than 60% of the total annual malaria cases among children under five occurring during this period [4]. As in the Sahel, the peak malaria transmission season coincides with the rainy season. Mathematical modelling conducted by the Swiss Tropical and Public Health Institute suggests that SMC using SPAQ in Karamoja at 80% coverage could reduce malaria cases and avert deaths in children under 5 years by reducing malaria prevalence from 20 to 5%, and incidence from 1500/1000 children to an average of < 100/1000 children over a three-year period [19]. These findings have prompted the MOH through the Uganda national malaria control and elimination strategic plan (UMRESP) of 2021–2025 to recommend a number of studies to assess whether SMC could be an acceptable, feasible and impactful malaria intervention to provide a substantial protection against malaria attack among children 3–59 years in this region during the peak season [20, 21]. This study was conducted by the NMCD and Malaria Consortium to investigate the protective effectiveness of SMC using SPAQ among children aged 3–59 in the Karamoja sub-region.

## Methods

### Study settings

**Fig. 1:**
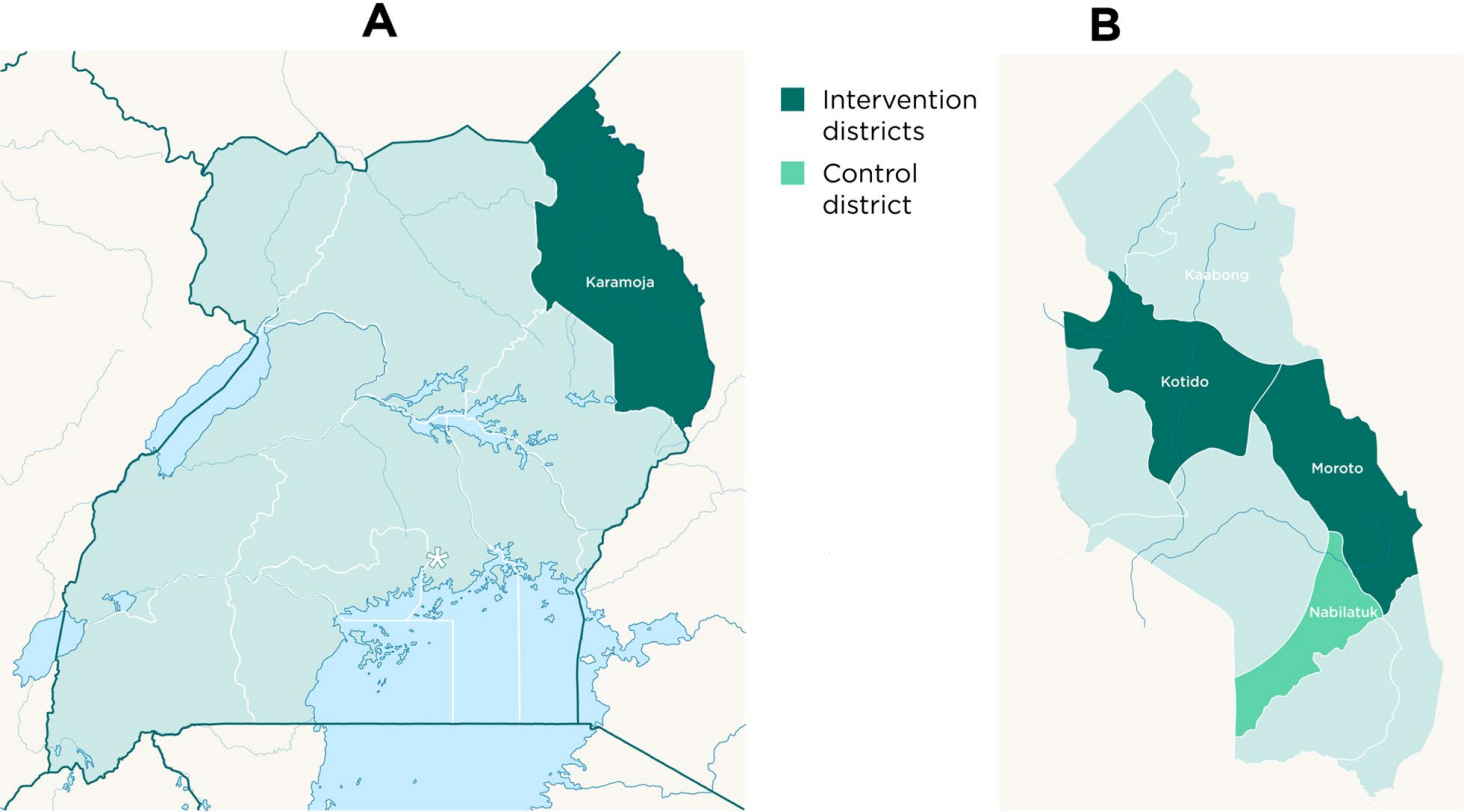
Maps showing Karamoja region in Uganda (**A**) and the study districts in Karamoja region (**B**)

This study was carried out as part of a larger implementation research project in three districts (Fig. 1). Five monthly cycles of SMC using SPAQ targeting 85,000 children were implemented in Kotido and Moroto with an average of 83,300 (98%) of these receiving monthly doses between May and September 2021. SMC was distributed door-to-door by community health workers also known as Village Health Teams (VHTs) in Uganda. A third district, Nabilatuk, served as a control where SMC was not implemented (Fig. 1). Standard malaria care was provided in all three study districts. The study team intervened to strengthen malaria case management, surveillance, community engagement and sensitization to malaria control interventions in all study districts. The districts share the same climatic conditions and have similar malaria transmission intensity. The populations of the districts have comparable demographic features.

### Study design

A two-arm quasi-experimental, open-label prospective nRCT was conducted between May and September 2021. The nRCT was part of a larger effectiveness-implementation hybrid type 1 study [22–24]. Its primary aim was to evaluate the effect of SMC in protecting children 3–59 months against malaria episodes over the five-month study period.

### Sampling strategy and study population recruitment

Health facilities served as the entry point for enrolment into the study population. A three-stage sampling strategy was deployed; At the first stage, only health centre level three and above in the study districts were purposively selected. In the second stage, eight villages with similarly high numbers of malaria cases were selected in each health facility catchment area in each district. This was based on malaria data for the same period of the preceding year extracted from health facilities’ outpatient registers. This data was then used to calculate malaria attack rates among children aged 3–59 months based on numbers of confirmed cases presenting at health centres divided by their catchment area populations. In the control district, communities bordering the intervention districts were not selected to avoid ‘leakage’ across district boundaries. In the third stage, all households in each of the eight selected villages were enumerated and twenty-five households with at least one SMC-eligible child were selected in each village using systematic sampling. One SMC-eligible child was then randomly selected from each household. This resulted in a sample of 200 children per district or 600 across the three study districts. To ensure adherence to the full three-day SPAQ regimen, children enrolled into the study in the intervention districts received all doses as directly observed therapy under the supervision of a VHT distributor. In the event of death of child under study, WHO’s verbal autopsy tool was applied to determine the cause of death [25]. SMC eligibility was defined according to the standard SMC protocol.

### Inclusion

All children aged 3–59 months at the time of sampling which took place 1–2 weeks before the first cycle of SPAQ administration and residents in the intervention and control districts were eligible for inclusion. Children with all levels of malnutrition including the severe forms were enrolled and received SPAQ if they were stable and able to take medicines orally; and not on other contraindicated medicines such as those containing sulpha. Unstable children with danger signs were referred to the health facilities for stabilization and further management. Once stabilized, the trained health workers at the facilities would determine an appropriate time to offer SPAQ for protection against malaria*.*

### Exclusion

- All children who were confirmed to be visiting the districts or who were sick or tested positive for malaria at enrolment stage, were excluded to participate in the five months’ study follow-up.
- Children below three and above 59 months
- Acute febrile illness or to severely ill children unable to take oral medication.
- Any child receiving cotrimoxazole prophylaxis.
- Allergy to any sulpha containing drug such as cotrimoxazole, bactrim, SP and AQ.

### Selection of study team, enrollment and follow up of children in the cohort

All children were enrolled on the first visit of the study team to each household. A short survey was administered to collect individual- and household-level data and confirm their eligibility in line with the set study inclusion and exclusion criteria. In a nutshell, children with confirmed malaria at the time of enrolment were excluded from the study participants. However, if an enrolled child develops malaria in subsequent follow-up rounds, they were treated with standard first line malaria treatment- Artemisinin-based combination therapy (ACT) and once declared free of malaria parasite by microscopy, they were permitted to receive SPAQ in the subsequent cycles of SMC. The study management team selected two clinical health workers with experience in malaria management from each health facility. They were trained and equipped with tools to conduct medical assessments for fever and confirmation of malaria. From May–September 2021, they conducted monthly follow-up of the 25 children enrolled in the study in each village. During each visit they assessed the children for sickness especially fever and conducted RDTs on those with fever or suspected to have malaria. Febrile children who tested negative with an RDT were referred to a health facility to validate the result using microscopy. In the intervention arm, children’s SMC status was ascertained. Only children who received full 3 day courses of SPAQ were considered to have received SMC. Data were collected using an electronic questionnaire using SurveyCTO version 2.71.

### Data management and analysis

Data were checked for consistency, cleaned in Microsoft Excel 2017, and analysed using Stata version 16 [25]. Malaria incidence (in terms of person-months) was calculated and the proportion of children experiencing single or multiple episodes of malaria during the follow up was determined. Children with no history of fever or who tested negative for malaria at a health facility or by VHTs were considered “not infected”. Those with malaria infections detected only once were considered “single episode” cases. Subsequent episodes were defined as those that occurred seven days after the previous episode with a confirmed negative malaria test by either RDT or microscopy or both.

Time to a positive malaria test diagnosis was calculated based on the date of enrolment into the study, and the date of positive malaria test diagnosis. ‘Failure’ was defined as visit to the health facility for suspected malaria and/or a confirmed case of malaria. Timing of this event was based on original date of health facility attendance during the study period. Time to event was measured in days. Kaplan–Meier curves [26] were used to describe probability of a positive malaria diagnosis. The Andersen-Gill extension of the Cox-proportional hazards regression model [27, 28] was used to compare risk of malaria from May to September 2021. The model generalizes Cox proportional hazards model to allow for analysis of recurrent events [29]. Data from the intervention group were compared to the control group. Significance level was set at 0.05.

### Ethical considerations

The study protocol and tools were approved by the Vector Control Division Research and Ethics Committee (VCDREC) and the Uganda National Council for Science and Technology (registration number: HS1182ES) which was approved on 10 February 2021. Written informed consent was obtained from the caregivers of recruited children.

## Results

### Baseline characteristics

**Fig. 2:**
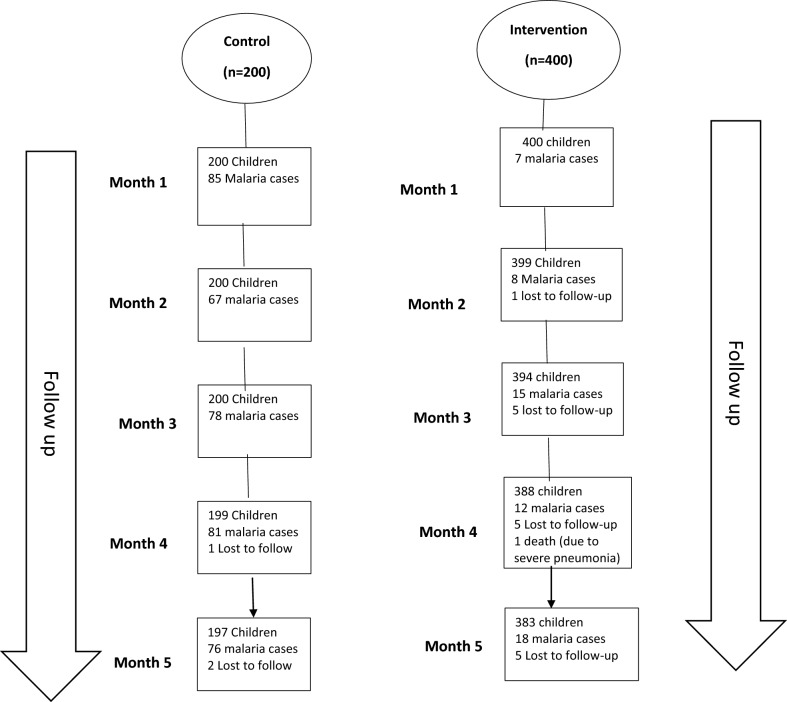
Schematic flow diagram of enrolment and follow-up

The two groups were comparable in terms of age, gender, parental education, mosquito net ownership and use. Household net ownership was high, with 98% of children were reported to have slept under a bed net the night before in both intervention and control districts (Table 1). None of the households sampled had received indoor residual spraying. Of the 600 children enrolled and followed up, 60 and 387 developed malaria over the 5 months among the intervention and control groups respectively (Fig. 2). One death was registered in the intervention group. This was investigated through verbal autopsy and cause of death determined to be severe pneumonia.

**Table 1:** Baseline characteristics of study subjects

Characteristic	Categories	Groups		P value
		Comparison (percent) n = 200	Intervention (percent) n = 400	
Age	3–5 Months	7 (3.5)	4 (1.0)	
	6–11 months	17 (8.5)	38 (9.5)	
	1 year	30 (15.0)	65 (16.3)	
	2 years	53 (26.5)	129 (32.3)	
	3 years	57 (28.5)	82 (20.5)	
	4 years	36 (18.0)	82 (20.5)	0.065
Sex	Female	92 (46.0)	211 (52.8)	
	Male	108 (54.0)	189 (47.3)	0.119
Parental education	Did not complete school	163 (81.5)	316 (79.0)	
	Primary education	16 (8.0)	32 (8.0)	
	Secondary education	16 (8.0)	28 (7.0)	
	Advanced education	5 (2.5)	24 (6.0)	0.300
Household mosquito net ownership	No	25 (12.5)	73 (18.3)	
	Yes	175 (87.5)	327 (81.8)	0.072
Child slept under a Long-lasting Insecticidal net (LLINs) the previous night	No	3 (1.7)	5 (1.5)	
	Yes	172 (98.3)	322 (98.5)	0.570
Household sprayed house (IRS)	No	199 (99.5)	399 (99.8)	
	Yes	1 (0.5)	1 (0.3)	0.556
Socioeconomic status	Highest (Fifth) quintile	23 (11.5)	90 (22.5)	
	Fourth quintile	53 (26.5)	70 (17.5)	
	Middle quintile	25 (12.5)	36 (9.0)	
	Second quintile	54 (27.0)	112 (28.0)	
	Lowest (First) quintile	45 (22.5)	92 (23.0)	0.004

### Use of standard malaria control interventions

During the 5 follow-up, the use of LLINs and other malaria interventions remained high in both arms (Table 2).

**Table 2:** Observations during 5 months of follow-up

Characteristic	Groups		Test statistic	p value
	Control (Percent)	Intervention (percent)		
LLINs				
No	111 (11.1)	373 (18.8)		
Yes	886 (88.9)	1609 (81.2)	28.7973	< 0.001
Child slept under net (previous night)				
No	19 (2.1)	86 (5.3)		
Yes	867 (97.9)	1523 (94.7)	14.5178	< .001
Indoor residual spraying				
No	992 (99.5)	1972 (99.5)		
Yes	5 (0.5)	10 (0.5)	Fisher’s exact	0.614

### Incidence of malaria

A total of 447 episodes of malaria infections were confirmed over the five-month study period, from 2979 observations. The incidence of malaria in the control group was higher throughout the study period, at 42.5% in month 1, 33.5% in month 2, 40.7% in month 3, 39.2% in month 4 and 39% in month 5. In the intervention group, the incidence always remained below 5% during the 5 month study period. The incidence rate of malaria after 1982 person months of observation was 3.0 cases per 100 person months in the intervention group, compared to 38.8 per 100 person months after 997 person months of observation in the control group (Table 3). The incidence rate ratio comparing the two groups was statically significant (p < 0.0001) at 0.078 (95% CI: 0.063–0.096) and the proportional hazards assumption of proportionality of hazards was met. Children in intervention districts had a 92.2 percent lower risk of developing confirmed malaria in the five-month follow-up versus those in the control district. Associations for other predictor variables, that is, child’s gender, educational status of parents of caregivers, mosquito net ownership, LLIN use, and indoor residual spraying were non-significant (Table 4).

**Table 3:** Incidence rates of malaria among children aged 3–59 months over 5 months of monthly SMC

Study arm	Person time of observation (months)	Number of episodes	Incidence-rate per 100 person-months	Incidence rate ratio (95% CI)	P-value
Intervention	1982	60	3.0	0.078 (0.063–0.096)	< 0.001
Control	997	387	38.8

**Table 4:** Crude and adjusted hazard ratios for different factors influencing a positive malaria diagnosis

Characteristic	Crude hazard ratio (95% CI)	p value	Adjusted hazard ratio (95% CI)	p value
Intervention versus control	0.7 (0.054–0.098)	< 0.001	0.0 (0.014–0.075)	< 0.001
Child’s gender	1.1 (0.837–1.381)	0.569		
Household mosquito net ownership	0.8 (0.607–1.124)	0.224		
Child slept under a mosquito net the night before	1.2 (0.628–2.198)	0.614	0.5 (0.111–1.904)	0.284
Indoor residual spraying of the household	1.5 (0.455–5.247)	0.486		
Education level of parents	0.9 (0.698–1.133)	0.343	1.1 (0.704–1.358)	0.895
Socio-economic status (Wealth index)	1.1 (0.834–1.089)	0.480	1.1(0.900–1.304)	0.396

### Malaria episodes over the follow up period

**Fig. 3:**
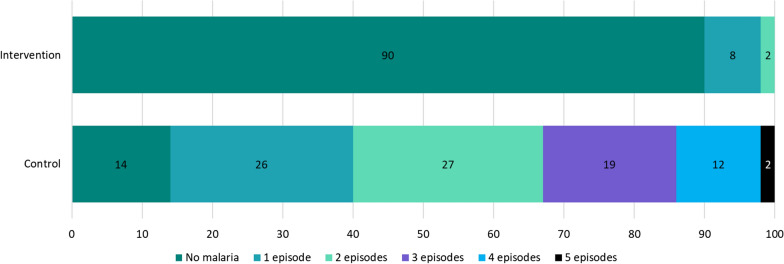
Proportion of malaria episodes observed during the follow-up period

In the intervention arm, 90% (361/400) of children did not experience a malaria episode, compared to 14% (29/200) in the control area. In the control arm, 85% (170/200) of children experienced at least one episode, 60% (119/200) had at least two episodes, and 2% had five malaria episodes over the five-month follow-up period (Fig. 3).

### Kaplan Meier plot comparing time to event among the two groups

**Fig. 4:**
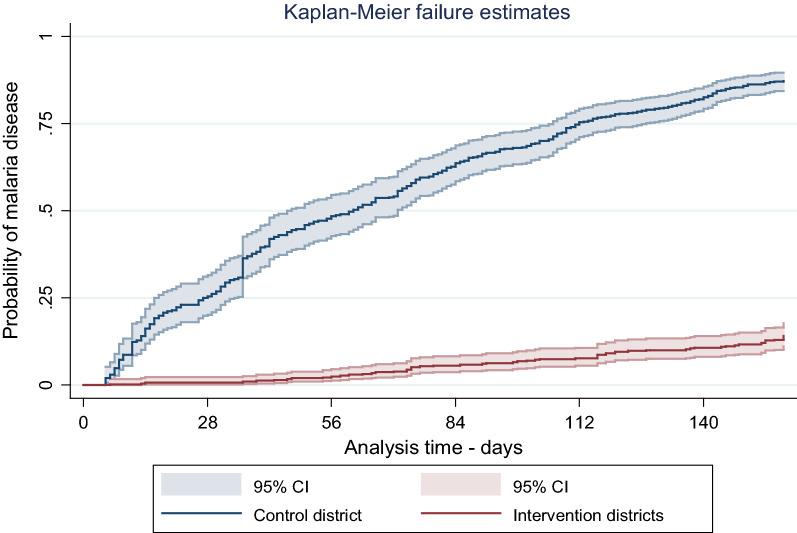
Kaplan Meir plots comparing malaria infection among intervention and control districts

In the curves below (log rank test statistic = 603.48; p < 0.0001), children in the control district experienced more failures. The rate ratio was 0.078 (95% CI 0.063–0.096) which corresponds to a protective effectiveness of 92% (95% CI 90.0–94.0) among children in the intervention area. The figure  shows that children in control group had higher probability of getting confirmed malaria than their counterparts in the intervention group over the five months follow-up period. (Fig. 4).

### Cox proportional hazards regression

The crude hazard ratio (HR) comparing malaria incidence in the intervention and control districts was 0.073 (95%CI 0.054–0.098; p < 0.001). None of the predictor variables had a significant association in univariable analysis. LLIN use the previous night, parental education and socioeconomic status were included in the multivariable cox proportional hazards regression model because of their historical protective influence against malaria. Indoor residual spraying was excluded because it was not being implemented in the study area. The hazard ratio for a positive malaria test remained statistically significant at 0.033 (95% CI 0.014–0.075; p < 0.001).

## Discussion

This study component assessed the protective effectiveness of SMC using SPAQ in a region known to have high prevalence of molecular markers of SP resistance. It was part of larger phase 1 SMC implementation research project with several other study components whose results will be presented elsewhere. Historically, the WHO recommended SMC with SPAQ to be implemented in the Sahel, where parasite resistance to SP is known to be low and SPAQ retains its therapeutic efficacy [11, 30]. This was due to a hypothesis that resistance to SP or AQ would reduce the efficacy of SMC in protecting children against malaria, although the relation between the degree of resistance and the effectiveness of SMC has not yet been clearly defined. The consolidated guidelines for malaria published by the WHO in 2022 no longer specify a therapeutic efficacy threshold for SPAQ, emphasizing the importance of local evidence in making decision on the deployment of SMC.

The study reported here is one of the first to provide evidence of the protective effect of SMC in the context of high parasite resistance. Effectiveness was high with SMC conferring a protective effect of 92%. Most children in the intervention arm (90%) did not develop any clinical malaria episode during the study period, compared to only 14% in the control group. Results from studies conducted in the Sahel region, an area of low SP resistance, demonstrated comparable protective effect sizes [7, 10, 31–33]. For instance, case–control studies across five countries in west and central African countries (Burkina Faso, Chad, The Gambia, Mali and Nigeria) found protective effects of SMC using SPAQ ranging from 72.9% to 98.3% [9]. The reduction in number of malaria episodes person-days of follow up among the children in the intervention group was greater than that found by a study conducted in Mali, which showed that the number of malaria episodes in treatment and control groups were 3.2 and 5.8 person-days of follow up [34].

This is not the only study which has demonstrated that chemoprevention with regimes containing SP in areas of high SP resistance provides protection against malaria. One study on IPTp conducted in 2007 showed that even in areas where a high proportion of *Plasmodium falciparum* parasites carry quintuple mutations, IPTp with SP remains effective in preventing the adverse consequences of malaria on maternal and fetal outcomes. The WHO recommends that SP should continue to be used for IPTp in areas of SP resistance [35, 36]. However, another recent study found that IPTp with SP in areas with high prevalence of SP resistance markers was not associated with reduced maternal malaria although the there was evidence of prophylactic effect against adverse pregnancy outcomes [37].

This study provides novel empirical evidence on the effectiveness of SMC using SPAQ in reducing malaria incidence. It substantiates evidence of the impact of SMC on malaria incidence and protective effect of SMC as demonstrated in similar studies in other African countries [9, 38, 39].

### Study strengths

The strengths of this study include the fact that, it was the first of its kind to investigate the protective effectiveness of SMC using SPAQ delivered for seasonal malaria chemoprevention in an area with documented resistance to SP in Uganda. The choice of appropriate intervention and control districts where subjects were drawn, with a relatively stable population led to a negligible loss to follow up. The study design ensured equal distribution of key variables such as age, gender, wealth status, housing structures, availability of LLINs, geographical location and malaria prevalence among both groups and therefore these variables had no effect as confounder. The number of children selected to be followed in both arms was appropriate and feasible. The all the children were closely monitored by VHTs who would conduct weekly home visits and a research team based at HF which would visit them monthly. Also, the caregivers were encouraged to urgently bring the child to health facility when they suspect that the child is sick and were provided with transport allowances to seek this care. There was verification of the diagnosis of malaria among the study subjects through the VHT registers and registers at various health facilities’ points of care where malaria testing and diagnosis was documented. Where the malaria RDT results seemed invalid, microscopy was applied. This eliminated possibilities of false diagnosis of a fever as malaria without confirmed laboratory results.

### Study limitations and mitigations

Non-randomized control study designs usually suffer from limited ability to guarantee the comparability of the intervention and control groups. However, this limitation was mitigated by: (i) collecting baseline data on all known confounders (i.e. characteristics with the potential to influence the outcomes) for both the intervention and control groups, (ii) to separate the effect of the intervention from other environment factors we collected data on the primary outcome on monthly basis in both groups, with one data point before and the start of the intervention, (ii) The follow-up visits on both the intervention and control groups were conducted at the same time, and (iv) same data collection procedure was used to collect data on both the intervention and control groups.

The study did not establish factors associated with breakthrough malaria infections among children receiving SPAQ. In the Karamoja region where SMC was deployed for the first time, understanding, and determining the factors associated with breakthrough infections could potentially guide decisions on the use of SPAQ as a chemotherapeutic agent for SMC, and scale up efforts for malaria control in the region. Further studies may be required to explore this.

## Conclusion

Even in areas with features indicative of high SP and AQ resistance, seasonal malaria chemoprevention with SPAQ provides excellent protective effect against malaria in the eligible targeted population, in this case children aged 3–59 months. These findings are very promising and could have policy implications. However, SP and AQ resistance remains a viable concern; hence, future studies should consider evaluating alternative SMC regimens in terms of effectiveness, feasibility, acceptability, and cost effectiveness. Alternative drug regimens could act as back up in case the chemoprevention efficacy SP and AQ declines over time, or they could be used in rotation with SPAQ to delay the development of resistance. Continuous and regular resistance marker monitoring is highly recommended track changes in these markers as SMC is being implemented in similar geographies especially at scale.

It is also likely that SP confers protection against clinical malaria episodes in combination with the partner drug AQ, even in areas with high prevalence of SP resistance markers. Further research may be needed in this context to investigate the chemoprevention efficacy and the long-term protective effect of SMC using SPAQ, over a defined period.

## Data Availability

The datasets are available from the corresponding author on reasonable request.

## Abbreviations

LLINs: Long-lasting insecticidal nets
SMC: Seasonal malaria chemoprevention
SP: Sulfadoxine-pyrimethamine
SPAQ: Sulfadoxine-pyrimethamine and amodiaquine
VHT: Village health teams
WHO: World Health Organization
